# Human S100A7 Induces Mature Interleukin1α Expression by RAGE-p38 MAPK-Calpain1 Pathway in Psoriasis

**DOI:** 10.1371/journal.pone.0169788

**Published:** 2017-01-06

**Authors:** Hu Lei, Xiangyun Li, Bo Jing, Hanzhang Xu, Yingli Wu

**Affiliations:** Hongqiao International Institute of Medicine, Shanghai Tongren Hospital/Faculty of Basic Medicine, Chemical Biology Division of Shanghai Universities E-Institutes, Key Laboratory of Cell Differentiation and Apoptosis of the Chinese Ministry of Education, Shanghai Jiao Tong University School of Medicine, Shanghai, China; Universitatsklinikum Hamburg-Eppendorf, GERMANY

## Abstract

Psoriatic keratinocytes express exaggerated levels of inflammatory cytokines, and show aberrant hyperproliferation and terminal differentiation in the pathogenesis of psoriasis. The antimicrobial protein hS100A7 (psoriasin) has been found highly expressed in psoriatic skin, but the mechanism and physiological function remain largely unknown. We observed that hS100A7 induces mature interleukin 1α (17kDa) expression in normal human epidermal keratinocytes, which is dependent on RAGE-p38 MAPK and calpain-1 as the inhibitors or knockdown of them completely decreased the expression of mature interleukin1α. Then, we proved mS100a7a15, mature IL-1α and calpain-1 were highly expressed in imquimod-induced psoriasis model and mouse IL-17a-neutralizing antibody treatment attenuated mS100a7a15 expression. At last, PD 151746 (calpain-1 inhibitor) treatment decreased epidermal thickness in imquimod-induced psoriasis model. Taken together, our results suggest that mature IL-1α induced by hS100A7 is via RAGE-p38 MAPK and calpain-1 pathway in keratinocyte and this mechanism may play an important role during psoriasis.

## Introduction

Psoriatic skin lesions major feature increased keratinocyte proliferation and abnormal differentiation.[1] The immunopathogenesis involves a dysregulated interaction between epidermal keratinocytes and infiltrating inflammatory cells.[2] The pro-inflammatory cytokine interleukin-1α is constitutively expressed by keratinocytes *in vivo* and has been shown to be expressed in psoriatic lesional skin.[3] Treatment of wild-type organotypic cultures with interleukin-1α was sufficient to induce hyperkeratosis in an *in vitro* model of lamellar ichthyosis.[4] IL-1 is likely to be an important mediator in the initiation and maintenance of psoriatic plaques and may represent an attractive therapeutic target.[5–7] It has been reported that proteolysis of IL-1α by calpain-1 results in a several-fold increase in bioactivity, which has nearly 50-fold higher affinity for IL-1R than full-length IL-1α.[8] Increased IL-1 activity is a hallmark of many chronic inflammatory conditions, including rheumatoid arthritis, diabetes, atherosclerosis, and psoriasis.[9, 10]

hS100A7 (psoriasin) belongs to the S100A family of Ca^2+^-binding proteins, it has been reported with many functions, such as antimicrobial,[11] chemotactic activity,[12, 13] and associated with some diseases, such as psoriasis,[14] skin tumors,[15, 16] atopic dermatitis,[17] and chronic rhinosinusitis.[18] These conditions are characterized by an inflammatory reaction, suggesting the role of hS100A7 in the regulation of inflammation. Our study for the first time reveals that hS100A7 induces mature IL-1α expression and other downstream signaling molecules *in vitro* and *in vivo*. It explains the mechanism of hS100A7 function in psoriasis, and provides a new target for the treatment of the disease.

## Materials and Methods

### Reagents

IL-1α antibody (SC-7929) was purchased from Santa Cruz Biotechnology. Antibodies directed against phospho-p38 MAPK (4511), p38 MAPK (8690), phospho-JNK (9251), JNK (9252), phospho-ERK (4370), ERK (4695), phosphor-AKT (9611) and AKT (4691) were purchased from Cell Signaling Technology. The antibody for human S100A7 (11141-RP02), soluble recombinant human S100A7 (11141-HNAE), IL17A (12047-HNAE), IL-22 (13059-HNAE), IL-33 (10368-HNAE), IL36γ (10124-HNAE) and TNFα (10602-HNAE) were purchased from Sino Biological Inc. The antibody against β-actin (ab8227), IL-1β (ab2105), RAGE (ab54741) and calpain-1 (ab28258) were purchased from Abcam. Antibody against mS100a7a15 (AP52284) was purchased from Abgent. The inhibitors including SB202190 (S7067), SP600125 (S5567), LY294002 (L9908) and caspase-8 inhibitor (C1230) were purchased from Sigma-Aldrich. PD151746 (S7424) was purchased from Selleck.

### Mice

All mice were housed and bred according to the Shanghai Medical Experimental Animal Care guidelines. Animal protocols were approved by the Institutional Animal Care and Use Committee of Shanghai Jiao-Tong University School of Medicine (SJTUSM IACUC).

### Cell culture

Normal human epidermal keratinocytes(NHEKs) were cultured in serum-free EpiLife medium (Cascade Biologics, M-EPICF-500) containing 0.6 mM Ca^2+^, 1× Epilife^®^ defined growth supplement (EDGS), 50 U/mL penicillin, and 50 μg/mL streptomycin under standard culture conditions.

### RNA interference and RNA analyses

The siRNA against p38, RAGE and calpain-1 are shown as follows: si-p38 MAPK 5’-AUGAAUGAUGGACUGAAAUGGUCUG-3’, si-RAGE: 5’-GAACTGAATCAGTCGGAGGAA-3’, si-calpain-1 5’-AAGAAGTTGGTGAAGGGCCAT-3’. Total RNA was isolated from mouse skin or cells by using RNAiso Plus (Takara, 9108), 500 ng total RNA was used for reverse transcription by PrimeScript^®^ RT reagent Kit (Takara, DRR037A). Quantitative Real-time PCR specific primers as shown as follows: IL-1α Forward 5’-GTGCTCAAAACGAAGACGAACC-3’ Reverse 5’-CATATTGCC ATGCTTTTCCCAGAA-3’, IL-1β Forward 5’-ACAGATGAAGTGCTCCTTCCA-3’ Reverse 5’-GTCGGAGATTCGTAGCTGGAT-3’, calpain-1 Forward 5’- AATTCCTCCAAGACCTATG-3’ Reverse 5’-TCCATCCACAATGAACTG-3’, GAPDH Forward 5’- CTTAGCACCCCTGGCCAAG-3’ Reverse 5’-TGGTCATGAGTCCTTCCACG-3’, mS100a7a15 Forward 5’- ACATCACGGACTGGCAGAACGT-3’ Reverse 5’-AGGATGTCGTGGAACTGGTCAG-3’, mIL-1α Forward 5’- GTGTTGCTGAAGGAGTTG-3’ Reverse 5’- ATGTGAAGTAGTTCTTAGAGTTG-3’ m18S Forward 5’- TGGGTCCTGTAGATGGCATTG-3’ Reverse 5’-GCTCCAGAAGGCCCTCAGA-3’. The comparative ΔΔ^CT^ method was used to determine the quantification of gene expression. The target gene expression in the test samples was normalized to the endogenous reference 18S rRNAor GAPDH level and reported them as the fold difference relative to 18S rRNA or GAPDH gene expression.

### Western blot

2mm mouse skin taken from mouse back or cells were lysed by using RIPA buffer (pH 7.4) containing protease inhibitor cocktail (Roche, 04693116001) and then were sonicated on ice-cold water for 15min. Protein concentrations of the extracts were measured by BCA^™^ Protein Assay Kit (Pierce, 23225) and equal amount of total protein from each sample was separated with SDS-PAGE and then transferred to nitrocellulose membrane followed by probing with the indicated antibodies.

### Cytokine release assay

NHEKs were plated, adhered overnight. Different concentrations of hS100A7 were added and incubated for 5 hours. Supernatants were clarified and cytokines assayed by ELISA (PeproTech, 900-T11).

### Imiquimod model of skin inflammation

10-week-old BALB/C mice were given 25 mg of imiquimod (InvivoGen, tlrl-imqs) in the shaved back as previous described [19]. 10 mg of PD151146 was i.p. injected 1 day before 25 mg of imiquimod was used to induce psoriatic skin. On day 5, mice were euthanized and back tissues were collected for immunohistochemical evaluation or mS100a7a15 and IL-1α mRNA or protein analysis.

### Immunohistochemistry and H&E staining

Skin samples were fixed with 4% paraformaldehyde for 2 days, then dehydrated through a graded series of ethanol and embedded in paraffin. Sections were cut and stained with haematoxylin-eosin (H&E). Immunohistochemistry was done as previously reported.[20]

### *In Vivo* IL-17a neutralization

100 μg of monoclonal mouse IL-17a antibody (R&D, MAB421) was intradermally injected into mouse back skin 24 hrs before experiment. Then imiquimod was injected, mouse skin was taken for analysis of mS100a7a15 expression 3 days later.

### Statistical analysis

Two-tailed t-test was used to determine significances between two groups. The significances among multiple groups were determined by One-way ANOVA with GraphPad 5 (San Diego, CA). For all statistical tests, we considered *P* values <0.05 to be statistically significant.

## Results

### hS100A7 induces mature IL-1α expression in normal human epidermal keratinocytes

**Fig 1:**
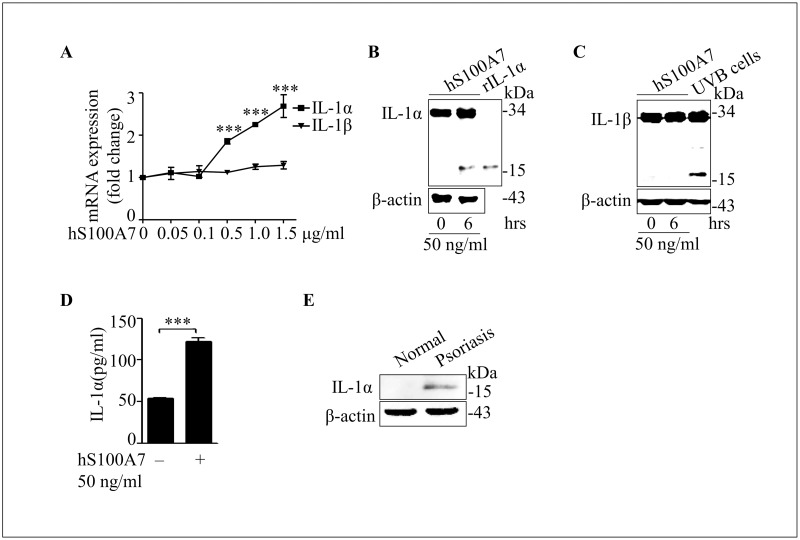
hS100A7 induces mature IL-1α expression in normal human epidermal keratinocytes. **(A)** IL-1α and IL-1β mRNA levels were measured by real time PCR after incubated with indicated concentrations hS100A7 at 6 hours. **(B)** Immunoblot of IL-1α treated with hS100A7 (50 ng/ml) at 6 hours or recombinant IL-1α protein (30 ng) by western blot in NHEKs. **(C)** Immunoblot of IL-1β treated with hS100A7 (50 ng/ml) at 6 hours or irradiated with broad-band UVB 4 mW/cm^2^ by western blot in NHEKs. (**D**) NHEK cells were incubated with hS100A7 (50 ng/ml) and concentrations of IL-1α in the supernatants were determined by ELISA after 5 hours. (**E**) Psoriatic epidermis was extracted by RAPI lysis buffer, IL-1α protein level was determined by western blot. All data are representative of three independent experiments with n = 3 and are means ± SEM. *P* values were determined by two-tailed t test. *** *P*<0.001.

IL-1α processing by multiple immune-related proteases can act as a switch to enhance the proinflammatory properties of this cytokine.[21] In our study, IL-1α and IL-1β mRNA levels were measured by real time PCR. The results demonstrated that hS100A7 treatment in keratinocyte induced IL-1α mRNA expression, but it can’t induce IL-1β mRNA expression (Fig 1A). IL-1α (17 kDa), not IL-1β (17 kDa), is induced by the treatment of hS100A7 in normal human keratinocytes (Fig 1B and 1C). The concentration of IL-1α in cell supernatant is also increased after hS100A7 treatment (Fig 1D). We also show that mature IL-1a is increased in psoriatic epidermis (Fig 1E). These data demonstrate that hS100A7 induce mature IL-1α (17 kDa) production in keratinocytes.

### P38 MAPK and RAGE are critical to mature IL-1α production induced by hS100A7

**Fig 2:**
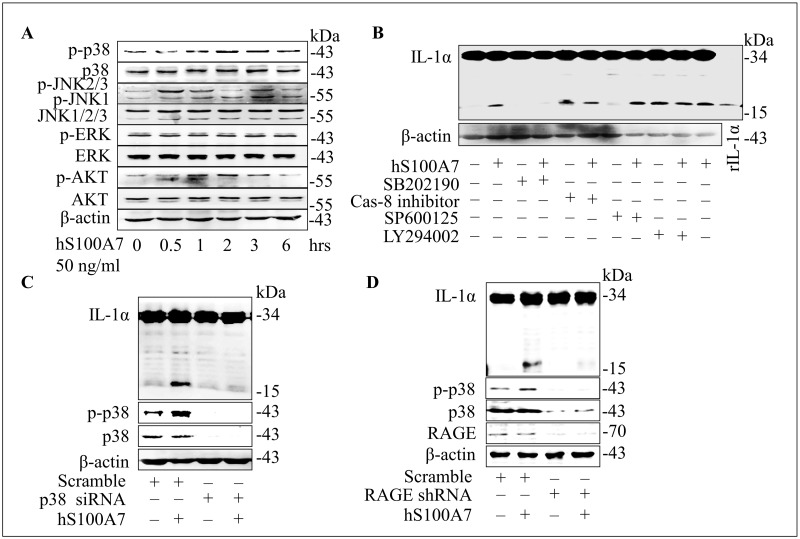
P38 MAPK and RAGE are critical to mature IL-1α production induced by hS100A7. **(A)** Immunoblot of proteins by different times treated with hS100A7 (50 ng/ml) in NHEKs. **(B)** Quantification of IL-1α protein expression in NHEKs treated with hS100A7 (50 ng/ml) in the absence or presence of SB202190 (p38 MAPK inhibitor, 15 μM), SP600125 (JNK inhibitor, 15 μM), caspase-8 inhibitor, (20 μM), and LY294002 (AKT inhibitor, 20 μM). The recombinant IL-1α protein (20 ng) showed as a positive control. **(C)** Mature IL-1α protein expression induced by hS100A7 after p38 MAPK was silenced by siRNA. **(D)** Mature IL-1α protein expression and p38 activation induced by hS100A7 after RAGE was silenced by siRNA. Each experiment was repeated as least three times.

The MAPK kinases, involved in the pathogenesis of psoriasis, control several important functions within the cell, such as cell proliferation, differentiation, gene expression, and apoptosis in keratinocytes.[22, 23] AKT-mediated signaling is functionally involved in keratinocyte transformation, differentiation and proliferation.[19, 24, 25] hS100A7 treatment activates p38 MAPK, JNK and AKT, but not ERK kinase (Fig 2A). We thereby used inhibitors of those pathways to treat undifferentiated NHEKs in the presence or absence of hS100A7. Among these inhibitors, p38 MAPK kinases inhibitor (SB202190) significantly blocked the effect of hS100A7 on mature IL-1α production, but this was not blocked by caspase-8 inhibitor, JNK inhibitor SP600125 and AKT inhibitor LY294002 (Fig 2B). P38 MAPK function was identified by specific siRNA, as knockdown of p38 MAPK expression blocked mature IL-1α production induced by hS100A7 (Fig 2C). It has been reported that extracellular hS100A7 binds to the transmembrane receptor RAGE, and RAGE can activate MAPK signaling pathway.[26, 27] After knockdown RAGE by siRNA, keratinocytes were treated with hS100A7 (50 ng/ml) for 24 hours. The results showed that hS100A7-induced p38 activation and mature IL-1α expression were depending on RAGE (Fig 2D). All these data suggest that hS100A7 binds to RAGE and actives p38 MAPK, which is involved in mature IL-1α production.

### Proteolysis of IL-1α induced by hS100A7 is dependent on calpain-1

**Fig 3:**
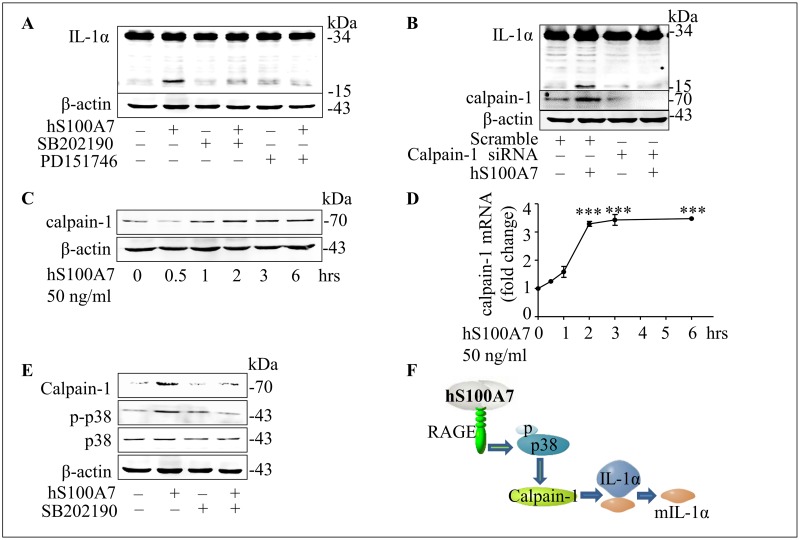
Proteolysis of IL-1α induced by hS100A7 is dependent on calpain-1. **(A)** Quantification of IL-1α protein expression treated with hS100A7 (50 ng/ml) in the absence or presence of SB202190 (p38 MAPK inhibitor, 15 μM), PD151746 (calpain-1 inhibitor, 15 μM) in NHEKs. **(B)** Mature IL-1α protein expression induced by hS100A7 at 6 hours after calpain-1 was silenced by siRNA. **(C and D)** Calpain-1 protein and mRNA expression determined by westernblot at different time points after treated with hS100A7 (50 ng/ml) in NHEKs. **(E)** Calpain-1 expression after blocking p38 MAPK activity by SB202190 (15 μM) in NHEKs. **(F)** The model shows that hS100A7 induces mature IL-1α expression via RAGE-p38 MAPK-calpain-1 signal pathway. All data are representative of three independent experiments with n = 3 and are means ± SEM. *P* values were determined by two-tailed t test. *** *P*<0.001.

Calpain-1, highly expressed in psoriasis, promotes restricted proteolysis of IL-1α to produce a 17 kDa protein.[28, 29] In our study, mature IL-1α production induced by hS100A7 can be blocked by calpain-1 inhibitor PD151746 in NHEKs (Fig 3A). Then we knocked down calpain-1 expression in NHEKs by specific siRNAs and detected IL-1α (17kDa) expression by western blot (Fig 3B), the results indicated that calpain-1 cleaved IL-1α (33 kDa) to IL-1α (17 kDa) in our experiments. Next, calpain-1 mRNA and protein expression are induced by hS100A7 at different times (Fig 3C and 3D). Both p38 MAPK and calpain-1 can induce mature IL-1α production, so we found that p38 MAPK activity blocked by SB202190 abolished calpain-1 expression induced by hS100A7 (Fig 3E). These data demonstrated hS100A7-induced mature IL-1α expression was depended on RAGE-p38 MAPK—calapin-1 pathway (Fig 3F).

### mS100a7a15, calpain-1 and mature IL-1α are induced in IMQ-induced psoriasis model

**Fig 4:**
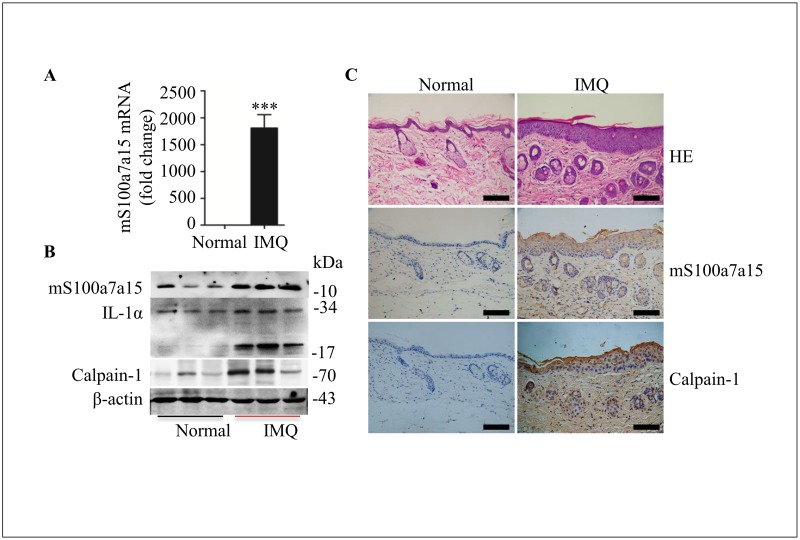
mS100a7a15, calpain-1 and mature IL-1α are induced in IMQ-induced psoriasis model. **(A)** Quantification of mS100a7a15 mRNA expression of skin from normal and imiquimod-induced psoriatic mouse backs at 5 days. **(B)** Immunoblot of mS100a7a15, IL-1α and calpain-1 in skin extracts from normal and imiquimod-induced psoriatic mouse backs. **(C)** HE staining and immunohistochemical analysis of mS100a7a15, calpain-1 in skin tissues as described in fig 4A. The scale bars represent 100 μm. All data are representative of three independent experiments with n = 6 and are means ± SEM. *P* values were determined by two-tailed t test. *** *P*<0.001.

Application of imiquimod on mouse skin results in the influx of various cells of the immune system, as well as hyperplasia of the epidermis, and IMQ-induced psoriasis-like skin inflammation in mice is mediated via the IL-23/IL-17 axis and IL-1R1/MyD88 signaling.[30–32] The psoriasis model was made as previously reported,[19] mS100a7a15 mRNA level was highly expressed in IMQ-induced skin tissue (Fig 4A). Also, mS100a7a15, calpain-1 and mature IL-1α protein levels were induced in psoriasis-like skin compared to normal skin (Fig 4B). Then mS100a7a15 and calpain-1 positive cells in IMQ-induced psoriasis skin are higher than that in normal skin by immunohistochemistry (Fig 4C). All the data mean that mature IL-1α, mS100a7a15 and calpain-1 are positive expressed in IMQ-induced psoriasis mouse model.

### PD151746 ameliorates epidermal hyperplasia in IMQ-treated mice

**Fig 5:**
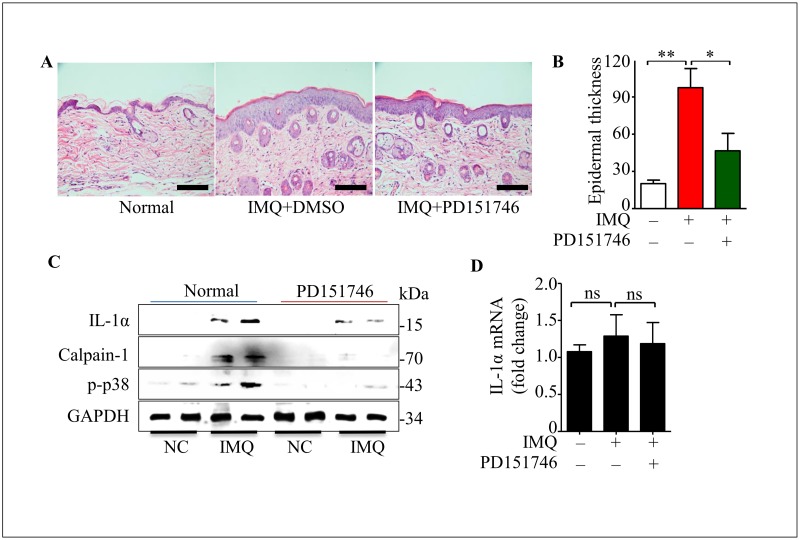
PD151746 ameliorates epidermal hyperplasia in IMQ-treated mice. **(A)** HE staining of skin in wild-type mouse treated by imiquimod (25 mg) in the absence or presence of PD151746 (10 mg) at 5 days. The scale bars represent 100 μm. **(B)** Epidermal thickness was measured by Image J. **(C)** Protein expression on mouse back skin as described in fig 5A by westernblot at 5 days. **(D)** IL-1α mRNA level was measured by real time PCR as shown in fig 5A. All data are representative of three independent experiments with n = 6 and are means ± SEM. *P* values were determined by one-way ANOVA. n.s., no significance. * *P*<0.05, ** *P*<0.01.

PD151746 is an inhibitor of calpain protein, the IC50 value of calpain-1 is 260 nM, with a 20-fold higher selectivity of calpain-2. Notably, PD151746 ameliorated psoriatic lesions and reversed epidermal hyperplasia (Fig 5A). Moreover, the PD151746 treatment caused a decrease of epidermal thickness on back (Fig 5B). Importantly, mature IL-1α and calpain-1 expression were decreased in the psoriasis-like lesions in the treatment of PD151746 (Fig 5C). However, IL-1α mRNA expression was not significantly changed (Fig 5D). Taken together, these data reveal that calpain-1 inhibition ameliorates psoriatic lesions *in vivo*.

### IL-17a induces hS100A7 or mS100a7a15 expression *in vitro* and *in vivo*

**Fig 6:**
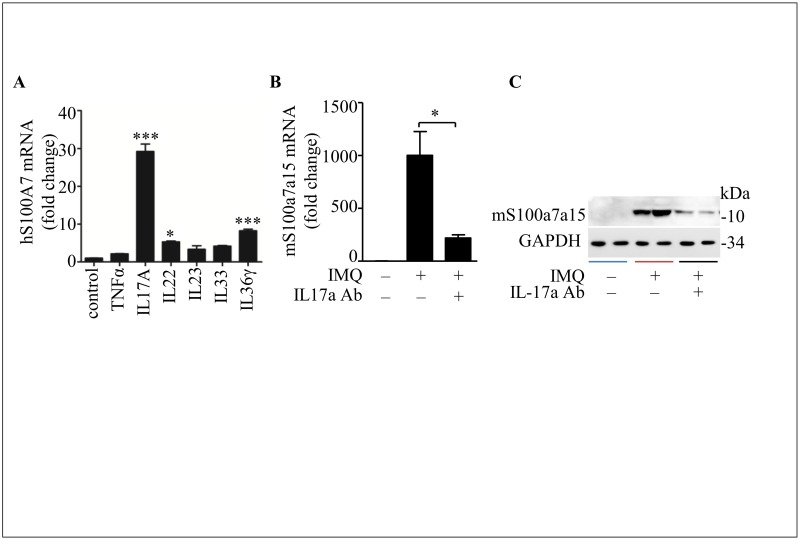
IL-17a induces hS100A7 or mS100a7a15 expression *in vitro* and *in vivo*. **(A)** Quantification of hS100A7 mRNA expression in undifferentiated NHEKs stimulated by different cytokines. TNFα = 50 ng/ml, IL17A = 50 ng/ml, IL22 = 50 ng/ml, IL23 = 15 ng/ml, IL33 = 20 ng/ml, IL36γ = 30 ng/ml. **(B and C)** mS100a7a15 mRNA and protein expression in mouse skin after IL-17a was neutralized by IL-17a-neutralizing antibody in imiquimod-induced mouse model. All data are representative of two independent experiments with n = 3–6 and are means ± SEM. *P* values were determined by two-tailed t test or one-way ANOVA. * *P*<0.05, *** *P*<0.001.

There are many inflammatory cytokines associated with psoriasis, it has been reported IL-17a plays a central role in the expression of psoriasis signature genes in keratinocytes.[33] In order to identify which cytokine is relevant to hS100A7 expression, we treated with NHEKs by different inflammatory cytokines. As shown in Fig 6A, hS100A7 is strongly induced by IL-17a, and IL-22 and IL-36γ also up-regulate the expression of hS100A7. Notably, IL-17a neutralizing antibody treatment in IMQ-induced psoriasis model decreased mS100a7a15 expression at mRNA and protein level (Fig 6B and 6C). All the data demonstrate that hS100A7 or mS100a7a15 expression is associated with IL-17a *in vitro* and *in vivo*.

## Discussion

This study demonstrated that hS100A7 treatment lead to mature IL-1α production in keratinocytes via RAGE-p38 MAPK-calpain-1 signaling. Several psoriasis-related cytokines, including IL-17a, IL-22 [34] and IL-36γ[35], could up-regulate hS100A7 expression in keratinocytes. IL-17a neutralizing antibody blocked mS100a7a15 expression in IMQ-induced psoriasis skin *in vivo*. Importantly, in a mouse model of psoriasis, calpain-1 inhibition improved epidermal hyperplasia, alleviated skin inflammation and reduced the expression levels of mature IL-1α and calpain-1. These results indicate that hS100A7 contributes to the pathogenesis of psoriasis by modulating keratinocyte activation.

Recent studies have reported that inflammatory cytokines can activate keratinocytes to produce cytokines and chemokines.[36] However, the mechanism of keratinocyte activation in psoriasis is not well understood. Inflammation is a driving force in psoriasis. It has been showed that IL-1α, IL-1β, IL-17A and IL-23 are important to psoriasis progress. However, it is interesting that hS100A7 induces mature IL-1α expression, not IL-1β. Pro-IL-1α can be cleaved to mature IL-1α by neutrophil elastase, granzyme B, chymase, and calpain-1, but neutrophil elastase and chymase also cleave pro-IL-1β. Granzyme B is commonly found in the granules of cytotoxic lymphocytes (CTLs), natural killer cells (NK cells) and cytotoxic T cells.[37] So we hypothesized that calpain-1 is essential to mature IL-1α production induced by hS100A7. Calpain-1 inhibition delays the wound healing, for reducing inflammatory cell recruitment and angiogenesis in the early stages of wound healing.[38] IL-1α plays an important role in chronic inflammation and it plays a pivotal role in triggering and sustaining the inflammatory process in a variety of disease indications. It is thought that targeting IL-1α holds significant promise as a unique therapeutic option for psoriasis and a variety of other diseases. IL-1R signaling is critical in skin inflammation. IL-23, IL-17 and IL-22 are markedly decreased in IL-1RI KO mice compared with WT mice which are treated by imiquimod.[39] Mostly, IL-1α deficiency results in impaired skin inflammation and leukocyte infiltration after UV exposure. Recombinant IL-1α enhances epidermal wound healing by stimulating fibroblast and keratinocyte growth and to induce collagen synthesis by fibroblasts in animal models, and IL-1α knockout mice also affect tissue repair and wound healing *in vivo*.[40, 41]

Human S100A7 is up-regulated in psoriatic skin lesions and is localized in epithelial cells and forms homodimers by non-covalent bindings. Increasing evidence suggests that hS100A7 plays critical roles in amplifying the inflammatory process in psoriatic skin, perpetuating the disease phenotype.[42, 43] In human cultured keratinocytes, hS100A7 also induce the expression of several differentiation markers, which are overexpressed in psoriatic skin.[44, 45] In our study, we confirmed that mS100a7a15 was overexpressed in psoriasis model skin. Mature IL-1α, not IL-1β, is induced by hS100A7 stimulation in keratinocytes, and mature IL-1α is also detected in *in vivo*. Human S100A7 expression is associated with epidermal thickness in psoriasis. Delphinidin was found to suppress hS100A7 expression in a 3D psoriatic skin equivalent model, and treatment with narrow-band UVB phototherapy induces a reduced production of hS100A7 in peripheral blood mononuclear cells in psoriatic patients.[43, 46] So, hS100A7 may serve as a potential therapeutic target for psoriasis with relatively few side effects.

## Conclusion

In conclusion, we demonstrate that hS100A7 induces mature IL-1α expression through RAGE-p38 MAPK-calpain-1 pathway and block mature IL-1α by calpain-1 inhibitor PD151746 can ameliorate epidermal hyperplasia in IMQ-treated mice. Human S100A7 is a critical player in the psoriatic maintenance phase, which may be considered as an additional and potential target molecule for psoriasis treatment.

## Abbreviations

hS100A7: human S100A7
IL-1: interleukin-1
MAPK: Mitogen Activated Protein Kinase
RAGE: Receptor for Advanced Glycation End products
